# Companies under stress: the impact of shocks on the production network

**DOI:** 10.1140/epjds/s13688-021-00310-w

**Published:** 2021-12-09

**Authors:** Róbert Pálovics, Primož Dolenc, Jure Leskovec

**Affiliations:** 1 Department of Computer Science, Stanford University, 353 Jane Stanford Way, Stanford, CA 94305 USA; 2 Bank of Slovenia, Slovenska cesta 35, Ljubljana, 1000 Slovenia

**Keywords:** Financial contagion, Shock propagation, Network evolution

## Abstract

In this paper we analyze the effect of shocks in production networks. Our work is based on a rich dataset that contains information about companies from Slovenia right after the financial crisis of 2008. The processed data spans for 8 years and covers the transaction history as well as performance indicators and various metadata of the companies. We define sales shocks at different levels, and identify companies impacted by them. Next we investigate stress, the potential immediate upstream and downstream impact of a shock within the production network. We base our main findings on a matched pairs analysis of stressed companies. We find that both shock and stress are associated with reporting bankruptcy in the future and that stress foremost impacts the future sales of customers. Furthermore, we find evidence that stress not only results in performance losses but the reconfiguration of the production network as well. We show that stressed companies actively seek for new trading partners, and that these new links often share the industry of the shocked company. These results suggest that both stressed customers and suppliers react quickly to stress and adjust their trading relationships.

## Introduction

Multimodal temporal links between companies result in the complex structure of today’s economy. Companies rely on the input and output of their trading partners. These linkages between suppliers and customers form the *production network*. The network consists of nodes (companies) with directed edges that define supplier and customer relations. This network is characterized by temporal dynamics since companies may change their suppliers and customers especially during turbulent times like the period after the 2008 global crisis.^1^ Due to unforeseen events companies experience *shocks*, and may be unable to fulfill their liabilities to others, and therefore impose stress on their upstream supplier and downstream customer partners. The effect of these individual company-level shocks [1, 2] in the ever-changing production network is still not completely understood.

Previous works cover results on economic networks of financial entities [3–14] and report characteristics of complex networks known in other domains, such as their power-law degree distribution [6] or the bow-tie structure [6, 15]. Although multiple theoretical models were proposed for financial contagion due to connectedness [16], yet there is limited empirical validation [3, 11, 12, 16] and the underlying network is often roughly estimated without empirical data on the links [3]. Promising theoretical models of production networks has been recently proposed [17, 18]. In contrast, there are only a few, mainly cross-sectional empirical studies addressing specifically company level supplier-customer relations and describing production networks [19–21]. These studies suggest that the network topology and the firms’ immediate connections have a crucial role in shock propagation. Fujiwara and Aoyama [19] provide a very detailed snapshot of a Japanese production network from 2006. In their analysis, the authors raise attention to the so-called “chain of bankruptcy”, meaning that when a firm goes into financial insolvency, its relations have elevated risk of going into bankruptcy. [20] examines the idiosyncratic risk in the context of firm interactions and highlight the tendency of companies to interact with firms with similar risk. According to Korniyenko et al. [21] the presence of “central players” in a specific product’s market represents significant risk for potential supply shocks since its crisis can destabilize a whole cluster of the production system.

As reviewed by [22], only a few studies focus on the propagation of the idiosyncratic shocks at the individual company level. A key assumption of these analyses is that suppliers impose significant output losses on their customers. Barrot et al. [2] show that natural disasters strongly influence not only the companies nearby within the region, but their customers as well, which consistently report sales growth drops. Studies that empirically investigate the immediate effects of shocks [2, 22–24] suggest sales growth as a main indicator of company performance.

Our work contributes to this stream of literature in several ways. We take advantage of a longitudinal dataset that makes it possible to analyzing the dynamics of the production network, especially during the times of the financial crises. In contrast to the earlier literature [2], we shift the focus from supply shocks, or sector level shocks [18, 22] to empirically investigate the consequences of drops in the sales of individual companies. Our work is based on a dataset covering the economic activities within Slovenia between 2008 and 2015, hence right after the global crisis of 2008 during the economic recession. The data includes timestamped transactions between the companies that we aggregate within each year. These yearly network snapshots reflect the changing customer-supplier relationships of companies. Furthermore, the data contains information on the industry sectors and the net sales of companies on a yearly basis. Our dataset is unique and different from other previously reported data [2, 19–21, 23, 24] since:

- The dataset is not limited to a single bank since all the information is available from the central bank of the country.
- The data contains all transactions within the country that have been sent through the Trans-European Automated Real-time Gross settlement Express Transfer system^2^ (TARGET2), which is the large-value international payments and settlement system for the Eurosystem, the eurozone’s system of central banks.
- The yearly balance sheet data indicates the performance of each company.
- The data is longitudinal and completely covers the 8 years between 2008 and 2015.

Relying on this information we aim to understand how shocked companies affect the behavior of their partners within the production network. We define shocks based on yearly sales changes available from the balance sheets and consider a company shocked if it experiences a sudden drop in its sales. We define the adjacent, immediate upstream, downstream and bi-directional partners of shocked companies *stressed* and analyze their behavior in the upcoming years.

**Figure 1:**
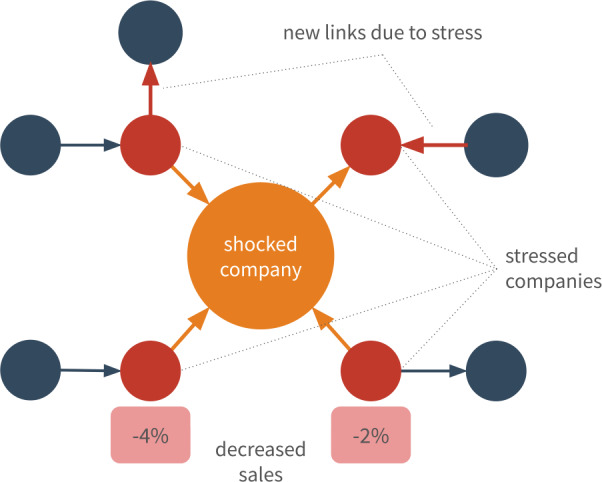
Results of a shock within the production network. Performance of customers in the neighborhood may drop within a year after the shock. In general, both customers and suppliers immediately respond and seek for new trading partners

More specifically, as illustrated in Fig. 1, we intend to examine the impact of shocked companies on the survival of their trading partners. We hypothesize that stressed companies experience a loss in performance during the years following the shocking event, and that stress may even contribute to their future bankruptcy. Furthermore, we assume that stress drives the reconfiguration of relations in the long run. Specifically, stressed companies react to the shocking event according to their relations with the shocked company: customers initiate new relationships with new suppliers, and suppliers initiate new links with customers accordingly.

In order to address these questions, we employ a matched pairs quasi-experimental design and match each stressed company to a company that does not experience shock or stress and has similar industrial, sales performance and network characteristics as the stressed one. We compare the future aspects of the stressed and control groups, and seek to answer if stress influences future economical performance or induces any changes within the production network.

Our results suggest that both shock and stress is associated with a higher chance of bankruptcy, and stress results in future decrease of sales performance. Furthermore, we find that stressed companies quickly adjust their relationships and seek for new trading connections suggesting that this behavior is key for resiliency against idiosyncratic shock propagation. The fact that new links often appear from the same industry as the shocked node indicate that these replace the previously shocked partner.

The rest of the paper is organized as follows. We provide a detailed description of the dataset in Sect. 2. Next in Sect. 3 we describe in detail the matched pairs design and the corresponding analyses. We present our results then in Sect. 4 and conclude them in Sect. 5.

## Dataset

**Figure 2:**
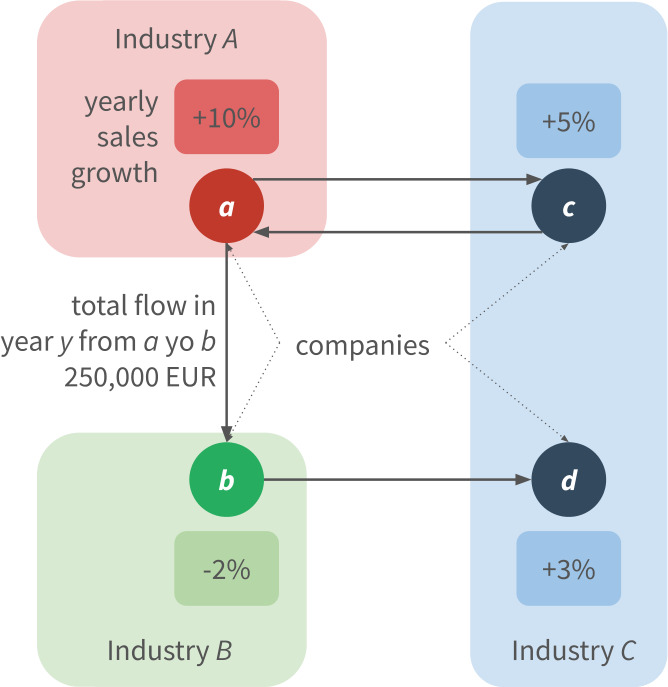
Structure of the processed dataset. We define customer-supplier relations on a yearly basis based on transaction history. Industrial sector and yearly sales information is also available for the companies

**Figure 3:**
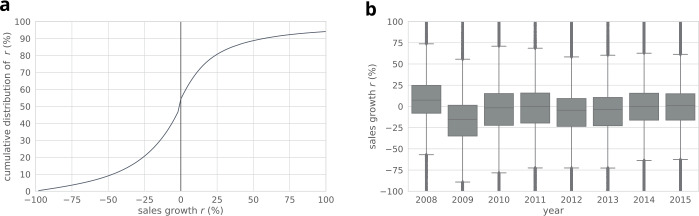
(**a**) Cumulative distribution of the yearly sales growth. (**b**) Yearly distributions of sales growth shown as box plots

Our work is based on a data of the economic activity of Slovenia (see Fig. 2). Briefly, the data covers yearly balance sheet information, daily transaction records and additional metadata of companies within the country. The yearly balance sheet information is available between 2000 and 2015. Following the concepts of [2, 23, 24], here we investigate *sales growth*, i.e., the yearly relative sales changes of companies. Note that we exclude from our analysis changes larger than 100%, which cover 6% of all the available records (see Fig. 3a). The median value of sales changes vary across the years (see Fig. 3b), showing a large drop in 2009. The data includes hierarchical industry sectors, defined by the national SKD system.^3^ We use the second level of this ontology, and since our analysis focuses on the production network, we remove companies related to financial services, or governmental activities resulting in 78 distinct sectors within the dataset (see Table 1). The metadata also indicates if a company went bankrupt anytime until 2015. In addition, we have access to all transactions recorded in the Trans-European Automated Real-time Gross settlement Express Transfer system (TARGET2) between 2008 and 2018. Companies of the study are required to send funds over 50k EUR via TARGET2, hence the data covers *all* of their large transactions. Since the balance sheet information is available on a yearly basis, we aggregate the transactions and define yearly directed networks of companies where for year *y* a directed edge $(A,B)$ exists between companies *A* and *B*, if *A* sent at least one transaction to *B* in *y*. We use the yearly aggregated networks to approximate customer-supplier relations.

**Table 1:** Industry categories sorted by their frequency within the data

% of companies	Name
18.12	Wholesale trade, except of motor vehicles and motorcycles
6.94	Architectural and engineering activities, technical testing and analysis
6.13	Specialised construction activities
5.57	Activities of head offices, management consultancy activities
5.19	Construction of buildings
4.91	Real estate activities
4.35	Manufacture of fabricated metal products, except machinery and equipment
3.98	Retail trade, except of motor vehicles and motorcycles
3.26	Land transport and transport via pipelines
3.14	Wholesale and retail trade and repair of motor vehicles and motorcycles
2.9	Computer programming, consultancy and related activities
2.2	Legal and accounting activities
1.81	Manufacture of machinery and equipment n.e.c.
1.61	Electricity, gas, steam and air conditioning supply
1.52	Food and beverage service activities
1.47	Civil engineering
1.37	Manufacture of rubber and plastic products
1.31	Warehousing and support activities for transportation
1.23	Scientific research and development
1.22	Advertising and market research
1.15	Manufacture of wood and products of wood and cork, except furniture, manufacture of articles of straw and plaiting mat.
1.06	Accommodation
0.97	Manufacture of furniture
0.89	Manufacture of food products
0.89	Repair and installation of machinery and equipment
0.87	Printing and reproduction of recorded media
0.81	Manufacture of other non-metallic mineral products
0.78	Manufacture of electrical equipment
0.71	Other professional, scientific and technical activities
0.7	Waste collection, treatment and disposal activities, materials recovery
0.7	Manufacture of computer, electronic and optical products
0.69	Travel agency, tour operator and other reservation service and related activities
0.64	Telecommunications
0.59	Publishing activities
0.58	Human health activities
0.57	Employment activities
0.49	Services to buildings and landscape activities
0.48	Manufacture of motor vehicles, trailers and semi-trailers
0.48	Office administrative, office support and other business support activities
0.45	Manufacture of chemicals and chemical products
0.45	Rental and leasing activities
0.43	Motion picture, video and television programme production, sound recording and music publishing activities
0.43	Crop and animal production, hunting and related service activities
0.41	Sports activities and amusement and recreation activities
0.38	Manufacture of textiles
0.38	Other personal service activities
0.38	Other manufacturing
0.37	Manufacture of basic metals
0.34	Manufacture of paper and paper products
0.31	Water collection, treatment and supply
0.3	Information service activities
0.28	Security and investigation activities
0.28	Programming and broadcasting activities
0.27	Manufacture of wearing apparel
0.25	Other mining and quarrying
0.23	Forestry and logging
0.21	Gambling and betting activities
0.18	Manufacture of other transport equipment
0.17	Repair of computers and personal and household goods
0.17	Creative, arts and entertainment activities
0.13	Manufacture of beverages
0.11	Postal and courier activities
0.09	Air transport
0.08	Residential care activities
0.08	Remediation activities and other waste management services
0.08	Water transport
0.08	Manufacture of leather and related products
0.08	Activities of membership organisations
0.06	Veterinary activities
0.06	Libraries, archives, museums and other cultural activities
0.05	Fishing and aquaculture
0.05	Sewerage
0.04	Manufacture of basic pharmaceutical products and pharmaceutical preparations
0.03	Social work activities without accommodation
0.02	Manufacture of coke and refined petroleum products
0.01	Mining support service activities
0.01	Extraction of crude petroleum and natural gas
0.01	Mining of coal and lignite

**Figure 4:**
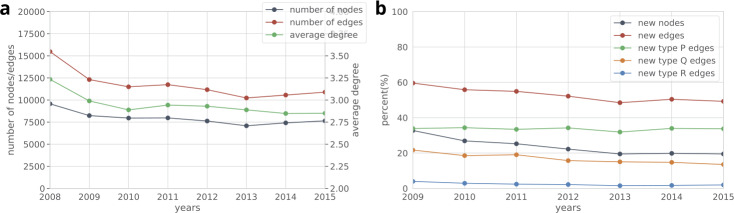
(**a**) Yearly graph statistics. The average degree, indicated in the right axis, is roughly constant while the overall activity decreases in the network. (**b**) Yearly fraction new edges and nodes in the production network. We distinguish between three new edge types, type P connects two already observed companies, type Q connects a newly observed company to an already seen one, type R connects two companies that have never been observed before

**Figure 5:**
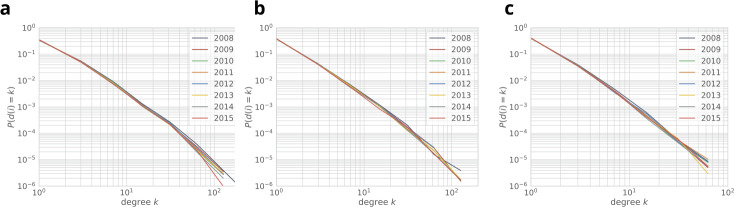
total degree (**a**), in-degree (**b**) and out-degree (**c**) distributions computed from yearly graph snapshots. While the underlying network changes, the power-law degree distribution is stable with exponent 2.27

Throughout our analyses we consider the period between 2008 and 2015 where all the listed information is available. Table 2 summarizes the properties of the processed dataset. In order to describe the production network, in Fig. 4a we plot yearly graph statistics. Note that the yearly number of edges declines, while the average degree only slightly decreases. In Fig. 4b we show for each year *y* the percent of nodes and edges that are first observed in year *y*. We distinguish three edge categories: a new edge can connect two already observed companies (P), it can connect a newly observed company to an already seen one (Q), and it can connect two companies that we never observed before (R). While several new edges appear each year, the fraction of R edges is low, i.e., most of the new edges connect to an already existing part of the network. In Fig. 5 we plot the degree distributions of the yearly networks. Despite the varying number of nodes and edges, the overall degree distribution does not change over the years. Note that the transitivity of these graphs are always below 0.005, indicating the lack of triangles. This property is specific to production networks, and it has been reported previously [19].

**Table 2:** Properties of the processed dataset

time span	2008–2015
number of companies	24,861
number of industry sectors	78
total number of transactions	478,620

## Methods

**Figure 6:**
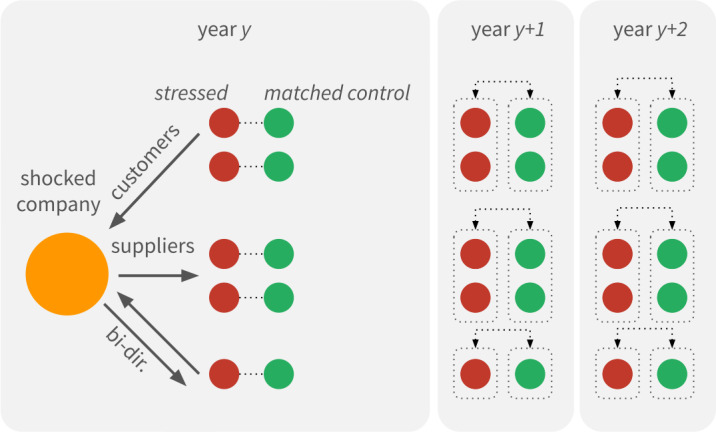
Concept of matched pairs analysis. We define partners of shocked companies stressed and match them to similar control companies. We compare the two groups during the two years following the original shock event

We follow previous related studies that use sales growth as a primary outcome variable [2, 23, 24]. We implement a time sensitive matched pairs based computational analysis in order to understand the effects of stress (see Fig. 6). Earlier studies report results on correlation based linear models [2, 23]. In contrast, our approach does not imply linear relationships. Furthermore, the variables of interest of this study are difficult to estimate overall, which can result in poorly fitted regression models.

### Definition of shock and stress

First, we define yearly sales growth of a company as

1 $$ r(c,y) = \frac{s(c,y) - s(c,y-1)}{s(c,y-1)}, $$

where $s(c,y)$ indicates the sales of company *c* in year *y*. Since company sales are non-negative, and we restrict our analysis for changes less than 100%, $-1 \leq r(c,y) \leq 1$. Based on this indicator we consider a company *c* in year *y* *shocked* if

2 $$ r(c,y-1)>0, \quad\quad r(c,y)< -\nu, $$

where shock level *ν* is a predefined parameter. In other words, we define a company shocked in a year if it suffers a great sales loss but operated without any decrease in sales in the previous year and hence showed no sign of suffering a shock. Note that once a company is shocked in year *s*, we exclude it from the analysis of the following years $y > s$.

We analyze the potential effect of these shocked companies on their network neighborhoods and define *stressed* companies (see Fig. 6). We consider company *t* stressed at level *ν* in year *y* if

- it has transaction record(s) from years earlier than *y* that links it to a company shocked at level *ν* in year *y*,
- initially does not indicate decreasing performance in year *y*, $r(t,y)>0$.

Since transactions reflect customer-supplier relations, we distinguish between stressed nodes depending on their relation to the shocked node. The successors of the shocked node are defined as suppliers, and the predecessors of the node are customers. We also distinguish bidirectional relations where cash flow was observed in both ways between the shocked and stressed companies. This categorization results in stressed suppliers, stressed customers and stressed bidirectional partners.

### Matching procedure

For each of the identified stressed company *t* we randomly select a matched control *c* company that

- has the same industry classification as the stressed one,
- has sales $s(c,y)$ in year that is similar to $s(t,y)$, and may differ up to 10%, i.e., $\vert s(t,y)-s(c,y) \vert /s(t,y)<0.1$,
- does not indicate decreasing performance in year *y*, $r(c,y)>0$,
- has never been shocked until year *y*,
- has similar in- and out-degree in year *y* to *t*.

For degree matching we match nodes with in/out-degrees that are at the same magnitude. This is particularly important since we have shown in Fig. 5 that both the in- and out-degree distributions are heavy tailed.

### Statistical analyses

First we investigate if shock events are related to bankruptcies and calculate the fraction of companies that eventually become bankrupt amongst all shocked and non-shocked companies. We repeat the analysis at shock levels between 20% and 80% and report the results of chi-square statistics. Next we compare stressed nodes to their matched control group. We calculate the fraction of bankrupt companies within the two groups at shock levels between 20% and 80% and report the results of chi-square statistics.

In order to put the bankruptcy related results into a wider context and estimate the effect sizes of the proposed shocked and stressed statuses, as a secondary analysis, we perform a logistic regression based classification on bankruptcy. We include all companies and aim to predict future bankruptcy based on company performance (sales and sales growth), the industry classification, the number of immediate relationships (suppliers and customers) and the status of the company (if the company ever suffered from stress, and shock). We are following a stepwise approach and in total, we run three models. The baseline model (STEP1) includes the industry classification, the mean total sales, the relative sales growth, the mean number of yearly customers (log transformed) and the mean number of yearly supplies (log transformed). In STEP2 we added the shock status (Yes/No, at 50% level) into the model in addition to the baseline predictors. Finally, (STEP3) the model includes stress (Yes/No,at 50% level) along with all the previous predictors. The continuous predictors are centered around the mean. We provide the statistical significance and parameter estimates of the individual predictors for the best model.

In order to assess how stress affects future sales performance, we compare the sales performance of the stressed companies to their control groups in the two years following the original shock event $(y+1,y+2)$. We conduct our analysis at shock levels between 20% and 80%. We perform independent t-tests between the stressed and control groups. In order to examine potential association between stress and the frequency of shocks, we perform chi-square tests.

We investigate network dynamics around the stressed and control nodes. We compare the number of new customers, suppliers and bidirectional relations that the stressed and control groups establish in one and two years following the original shock event. As presented in Sect. 2, the yearly customer-supplier networks have heavy tailed degree distributions. Hence we test the significance of these results with the non-parametric Kolmogorov–Smirnov test.

Finally, we ask if stressed companies potentially “replace” their shocked partners and find new connections within the industry sector of the shocked node. We investigate at 50% shock level the new suppliers of shocked customers and the new customers of shocked suppliers. We calculate the ratio of stressed nodes where the shocked neighbor(s) industry categories can be found within the new links initiated in followup years ($y+1$ and $y+2$).

## Results

**Figure 7:**
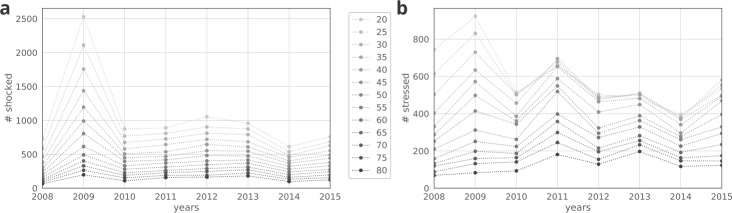
(**a**) Number of shocked companies per year at different shock levels. (**b**) Number of stressed companies per year at different shock levels

Overall, the economical crisis results in several shocked and stressed nodes, e.g., at 50% shock level there are roughly 500 shocked as well as stressed companies within each year following 2008 (see Fig. 7, Tables 3, 4). Our results show that companies are being shocked most prominently in 2009, which is consistent with the results of Fig. 3b, where 2009 appears to be the worst year in terms of sales indicating the burden of the 2008 financial crisis.

**Table 3:** Number of shocked companies per year at different shock levels

Year	Shock level												
	20	25	30	35	40	45	50	55	60	65	70	75	80
2008	728	585	466	380	316	276	231	197	161	130	111	88	65
2009	2531	2112	1757	1438	1195	991	805	615	491	402	330	265	198
2010	875	768	675	580	505	450	395	332	281	228	190	152	110
2011	890	809	727	643	537	469	418	370	311	261	229	190	157
2012	1054	903	811	721	646	554	482	402	347	290	247	191	164
2013	958	875	791	685	609	546	475	417	369	312	272	224	181
2014	612	539	483	455	404	367	324	280	240	199	161	133	98
2015	760	686	629	555	491	443	380	329	278	246	196	155	123

**Table 4:** Number of stressed companies per year at different shock levels

Year	Stress level												
	20	25	30	35	40	45	50	55	60	65	70	75	80
2008	743	614	503	403	331	288	252	210	158	133	119	88	69
2009	923	831	730	634	572	498	414	312	251	198	159	132	83
2010	514	502	503	457	386	361	344	262	224	187	164	141	93
2011	657	659	677	695	653	588	549	519	398	358	299	245	181
2012	504	492	474	484	464	409	323	295	272	215	196	155	129
2013	480	503	510	502	482	449	389	363	329	282	256	233	197
2014	396	378	379	369	341	296	277	261	226	192	163	147	117
2015	534	581	554	499	492	469	396	329	294	235	174	146	123

### Shock, stress and bankruptcies

**Figure 8:**
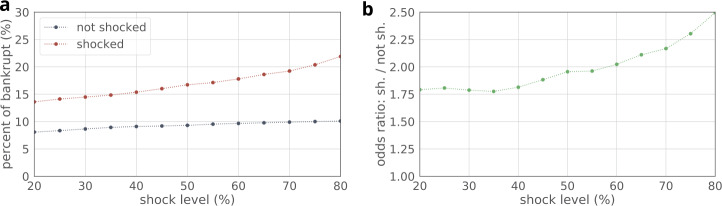
(**a**) Fraction of bankrupt companies among shocked and non-shocked ones. (**b**) Odds-ratio of bankruptcy. We run chi-square test per shock level and find all results significant with $p<0.001$

We address the question if shock events are related to bankruptcies. Figure 8 shows the fraction of companies that eventually become bankrupt within shocked and non-shocked companies. The results of the chi-square statistic shows that shocks and bankruptcy are significantly associated at all shock levels (all $p<0.001$). These translate to odds ratios (OR) around 2 (see Fig. 8b, e.g., at shock level 50% $\mathrm{OR} =1.95$, $p<0.001$). Hence for every company with at least 50% drop in performance, almost twice as many will be bankrupted in the following years as will without being shocked.

**Figure 9:**
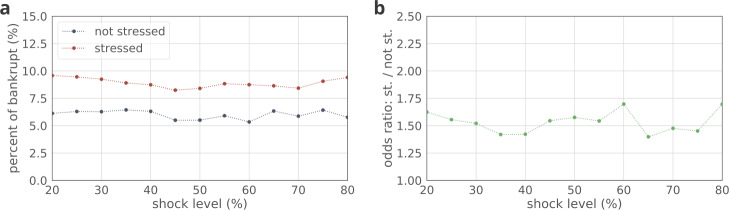
(**a**) Fraction of bankrupt companies among stressed and non-stressed ones. (**b**) Odds-ratio of bankruptcy. We run chi-square test per shock level and find all results significant with $p<0.001$

More importantly, stress is also significantly related to bankruptcy (see Fig. 9). The fraction of bankrupt companies differ by groups, stressed companies are more likely to be bankrupt then their matched controls (see Fig. 9a). The relationship is significant at all shock levels, and translates to odds ratios (OR) around 1.5 (see Fig. 9b). E.g., at shock level 50% $\mathrm{OR} =1.57$, $p<0.001$, meaning that the likelihood to be bankrupt is 1.5 times higher for a stressed node (defined by a 50% cutoff) than for a control node.

We continue the analysis of bankruptcies with an additional logistic regression based analysis. The pseudo-$R^{2}$ value of the baseline is fairly low (0.044) indicating that the task is hard in general. However, including shock improves the pseudo-$R^{2}$ score ($R^{2} = 0.055$), which is also increased by adding the stress ($R^{2} = 0.060$) into the model (STEP3). We provide the statistical significance of the predictors for the final, best performing model (STEP3) in Table 5. According to the results both the stress ($\beta = 0.428$, $p <0.001$) and shock ($\beta = 0.737$, $p <0.001$) status of the company is significantly associated with future bankruptcy. Moreover the company’s sales ($\beta = -0.275$, $p<0.01$) is also predictive of bankruptcy and companies with higher sales are less likely to go bankrupt. The number of customers ($\beta = 0.173$, $p <0.001$) is positively associated with the bankruptcy, while the number of suppliers does not significantly impact the outcome. The sales growth does not have a significant effect in the model. Some sectors of the industry categories seem to be significantly predictive of bankruptcy, which is in line with the previous literature showing that the production sectors are at higher risk of becoming bankrupt (for a detailed review of the sector based analysis see [22]). Altogether these results are in line with the findings of our match-paired experiment and further confirm the impact of both shock and stress on bankruptcy.

**Table 5:** Summary of the logistic regression analysis. Variables included in the final model of bankruptcy (pseudo-$R^{2} = 0.06$). Parameter estimates *β* with 95% confidence intervals (CI) are provided. In the table we report industry categories with significant impact in the model. $* p < 0.05$, ${{*}{*}} p < 0.01$, ${*{*}*} p < 0.001$

	*β*	std	*z*	*p*	CI: [0.025	0.975]
Intercept	−2.872	0.466	−6.16	<0.001∗∗∗	−3.786	−1.958
stress	0.428	0.058	7.332	<0.001∗∗∗	0.313	0.542
shock	0.737	0.058	12.616	<0.001∗∗∗	0.622	0.851
customers	0.173	0.027	6.503	<0.001∗∗∗	0.121	0.226
sales	−0.275	0.08	−3.445	0.001∗∗∗	−0.431	−0.118
Construction of buildings	1.405	0.472	2.976	0.003∗∗	0.48	2.33
Legal and accounting activities	−2.177	0.744	−2.927	0.003∗∗	−3.634	−0.719
Printing and reproduction of recorded media	1.171	0.512	2.286	0.022∗	0.167	2.176
Gambling and betting activities	1.389	0.62	2.241	0.025∗	0.174	2.604
Manufacture of wearing apparel	1.276	0.598	2.134	0.033∗	0.104	2.45
Employment activities	1.0952	0.539	2.032	0.042∗	0.039	2.152
Manufacture of paper and paper products	1.172	0.583	2.01	0.044∗	0.029	2.315
Food and beverage service activities	0.980	0.497	1.974	0.048∗	0.007	1.954

### Impact of stress on future sales performance

**Figure 10:**
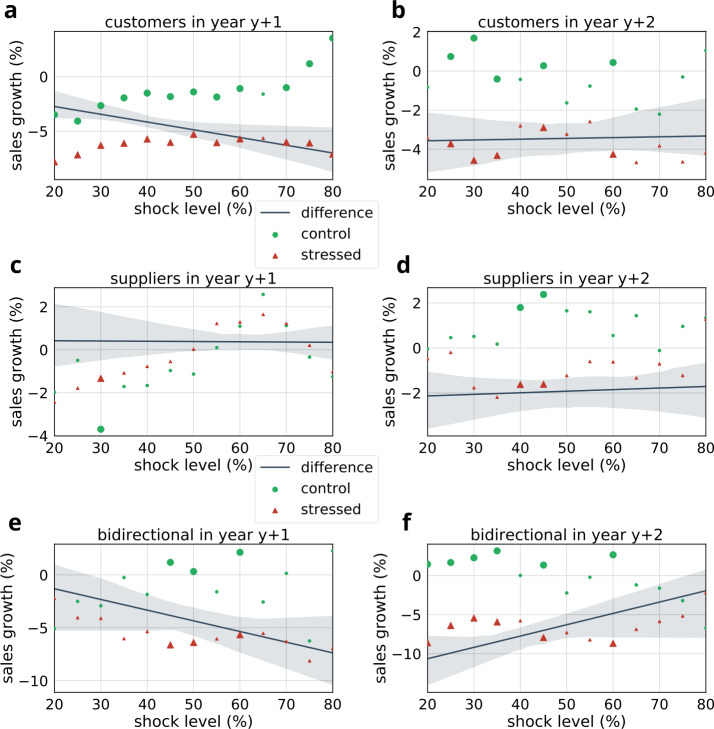
Comparison of mean sales growth between stressed and control groups at different shock levels. Large bullets indicate significant differences ($p<0.05$). We provide trendlines fitted on the differences between the groups. Results are shown separately for stressed customers (**a**, **b**), suppliers (**c**, **d**) and bidirectional partners (**e**, **f**). (**a**, **c**, **e**) indicate sales growth in the first year following the shock event ($y+1$), while (**b**, **d**, **f**) show the changes in the second year following the shock event ($y+2$). The corresponding statistics and sample sizes are listed in Table 6

We compare the future sales performance of stressed companies to their matched controls’ performance. Our results are summarized in Fig. 10 and Table 6. For stressed customers, the difference of performance is significant between stressed and control groups nearly at all shock levels (see Fig. 10a) in the follow-up year $y+1$. E.g., at 50% shock level, $t=2.51$, $p<0.012$. The fitted trend line suggests that the difference increases at larger shock levels. Similar, although not consistent and smaller differences can be observed in the second year after the original shock event (see Fig. 10b). In contrast to the customers, in case of stressed suppliers we have not identified any relevant trend in the group difference (see Figs. 10c–d). Finally, in case of bidirectional partners (see Fig. 10e–f) at the first year follow-up the results show a similar trend as seen in the case of stressed customers, however group differences are significant only for certain shock levels (40%–60%). The group differences are still very pronounced in the second year follow-up. We must note though that sample sizes for bidirectional partners are an order of magnitude lower than for customers or suppliers (see Table 6).

**Table 6:** Comparison of the sales growth between stressed and control groups at different shock levels. Results are shown separately for stressed customers, suppliers and bidirectional partners in the first and second years following the shock event. We provide sample sizes (*N*), group means and standard deviations, t-statistics and corresponding *p*-values of independent t-tests between the groups

Stress level drop in sales (%)	Edge type	Year	*N*	Stressed mean ± SD	Control mean ± SD	t-value	*p*-value
20	customers	1	977	−7.80 ± 28.19	−3.48 ± 26.97	3.46e + 00	5.48e − 04∗
25	customers	1	925	−7.16 ± 28.57	−4.07 ± 27.04	2.39e + 00	1.71e − 02∗
30	customers	1	919	−6.29 ± 28.17	−2.65 ± 28.26	2.77e + 00	5.75e − 03∗
35	customers	1	866	−6.10 ± 28.05	−1.94 ± 27.76	3.10e + 00	1.96e − 03∗
40	customers	1	792	−5.71 ± 26.56	−1.50 ± 28.32	3.05e + 00	2.34e − 03∗
45	customers	1	718	−6.02 ± 26.83	−1.83 ± 26.37	2.99e + 00	2.87e − 03∗
50	customers	1	614	−5.28 ± 27.57	−1.39 ± 26.66	2.51e + 00	1.21e − 02∗
55	customers	1	509	−6.04 ± 27.76	−1.86 ± 26.53	2.45e + 00	1.43e − 02∗
60	customers	1	408	−5.70 ± 28.56	−1.09 ± 25.00	2.45e + 00	1.44e − 02∗
65	customers	1	338	−5.63 ± 28.28	−1.59 ± 26.10	1.93e + 00	5.40e − 02
70	customers	1	299	−5.99 ± 28.15	−1.00 ± 24.01	2.33e + 00	2.00e − 02∗
75	customers	1	236	−6.08 ± 28.02	1.19 ± 27.88	2.82e + 00	4.98e − 03∗
80	customers	1	168	−7.09 ± 26.85	3.54 ± 25.76	3.70e + 00	2.49e − 04∗
20	customers	2	977	−3.43 ± 31.54	−0.83 ± 27.68	1.94e + 00	5.25e − 02
25	customers	2	925	−3.72 ± 31.70	0.74 ± 27.68	3.23e + 00	1.27e − 03∗
30	customers	2	919	−4.57 ± 31.66	1.69 ± 27.91	4.49e + 00	7.45e − 06∗
35	customers	2	866	−4.32 ± 32.38	−0.41 ± 27.72	2.70e + 00	7.01e − 03∗
40	customers	2	792	−2.80 ± 31.76	−0.43 ± 27.53	1.59e + 00	1.12e − 01
45	customers	2	718	−2.89 ± 32.14	0.27 ± 28.20	1.98e + 00	4.74e − 02∗
50	customers	2	614	−3.23 ± 32.85	−1.63 ± 25.77	9.51e − 01	3.42e − 01
55	customers	2	509	−2.58 ± 33.83	−0.77 ± 26.64	9.51e − 01	3.42e − 01
60	customers	2	408	−4.26 ± 35.42	0.44 ± 26.71	2.14e + 00	3.28e − 02∗
65	customers	2	338	−4.68 ± 35.59	−1.95 ± 27.60	1.11e + 00	2.65e − 01
70	customers	2	299	−3.83 ± 35.76	−2.21 ± 27.23	6.23e − 01	5.34e − 01
75	customers	2	236	−4.65 ± 35.27	−0.30 ± 29.34	1.46e + 00	1.46e − 01
80	customers	2	168	−4.17 ± 34.29	1.05 ± 25.22	1.59e + 00	1.13e − 01
20	suppliers	1	996	−2.43 ± 27.34	−1.98 ± 24.63	3.88e − 01	6.98e − 01
25	suppliers	1	928	−1.78 ± 26.79	−0.49 ± 25.11	1.07e + 00	2.84e − 01
30	suppliers	1	889	−1.33 ± 26.68	−3.70 ± 23.79	−1.98e + 00	4.82e − 02∗
35	suppliers	1	825	−1.08 ± 26.78	−1.71 ± 24.57	−4.99e − 01	6.18e − 01
40	suppliers	1	721	−0.77 ± 26.33	−1.66 ± 25.97	−6.47e − 01	5.18e − 01
45	suppliers	1	635	−0.54 ± 26.16	−0.97 ± 24.28	−2.98e − 01	7.65e − 01
50	suppliers	1	562	0.03 ± 25.31	−1.13 ± 24.60	−7.78e − 01	4.37e − 01
55	suppliers	1	483	1.23 ± 25.10	0.10 ± 24.67	−7.01e − 01	4.84e − 01
60	suppliers	1	420	1.29 ± 24.73	1.10 ± 22.64	−1.18e − 01	9.06e − 01
65	suppliers	1	378	1.65 ± 26.66	2.58 ± 24.06	5.02e − 01	6.16e − 01
70	suppliers	1	320	1.23 ± 27.05	1.13 ± 26.89	−4.84e − 02	9.61e − 01
75	suppliers	1	273	0.21 ± 26.59	−0.34 ± 27.20	−2.39e − 01	8.11e − 01
80	suppliers	1	184	−1.02 ± 23.55	−1.25 ± 26.26	−9.09e − 02	9.28e − 01
20	suppliers	2	996	−0.47 ± 29.74	−0.05 ± 24.21	3.52e − 01	7.25e − 01
25	suppliers	2	928	−0.20 ± 28.96	0.46 ± 24.76	5.26e − 01	5.99e − 01
30	suppliers	2	889	−1.76 ± 29.88	0.51 ± 25.73	1.72e + 00	8.57e − 02
35	suppliers	2	825	−2.19 ± 29.06	0.17 ± 24.76	1.77e + 00	7.63e − 02
40	suppliers	2	721	−1.62 ± 29.88	1.79 ± 27.13	2.27e + 00	2.32e − 02∗
45	suppliers	2	635	−1.61 ± 30.12	2.38 ± 26.01	2.52e + 00	1.17e − 02∗
50	suppliers	2	562	−1.22 ± 29.71	1.65 ± 26.95	1.70e + 00	9.01e − 02
55	suppliers	2	483	−0.60 ± 28.43	1.61 ± 25.51	1.27e + 00	2.04e − 01
60	suppliers	2	420	−0.62 ± 28.05	0.55 ± 24.84	6.39e − 01	5.23e − 01
65	suppliers	2	378	−1.33 ± 29.76	1.43 ± 25.85	1.36e + 00	1.74e − 01
70	suppliers	2	320	−0.70 ± 28.53	−0.11 ± 24.35	2.82e − 01	7.78e − 01
75	suppliers	2	273	−1.22 ± 29.87	0.96 ± 24.06	9.39e − 01	3.48e − 01
80	suppliers	2	184	1.28 ± 26.44	1.34 ± 24.17	2.33e − 02	9.81e − 01
20	bidirectional	1	159	−2.23 ± 26.23	−5.08 ± 22.37	−1.04e + 00	2.97e − 01
25	bidirectional	1	154	−4.04 ± 28.64	−2.50 ± 23.17	5.18e − 01	6.05e − 01
30	bidirectional	1	142	−4.10 ± 29.32	−2.91 ± 19.59	4.00e − 01	6.89e − 01
35	bidirectional	1	135	−6.01 ± 30.46	−0.25 ± 21.06	1.81e + 00	7.15e − 02
40	bidirectional	1	135	−5.35 ± 28.78	−1.84 ± 18.96	1.18e + 00	2.38e − 01
45	bidirectional	1	119	−6.61 ± 26.59	1.19 ± 23.80	2.38e + 00	1.79e − 02∗
50	bidirectional	1	102	−6.38 ± 26.31	0.32 ± 20.75	2.02e + 00	4.46e − 02∗
55	bidirectional	1	92	−6.04 ± 28.77	−1.59 ± 23.32	1.15e + 00	2.51e − 01
60	bidirectional	1	86	−5.65 ± 27.51	2.13 ± 20.33	2.11e + 00	3.63e − 02∗
65	bidirectional	1	77	−5.52 ± 26.50	−2.56 ± 23.30	7.37e − 01	4.62e − 01
70	bidirectional	1	69	−6.29 ± 28.49	0.15 ± 24.70	1.42e + 00	1.58e − 01
75	bidirectional	1	57	−8.12 ± 30.44	−6.24 ± 24.03	3.66e − 01	7.15e − 01
80	bidirectional	1	50	−6.93 ± 31.32	2.28 ± 25.85	1.60e + 00	1.12e − 01
20	bidirectional	2	159	−8.60 ± 30.35	1.45 ± 20.51	3.46e + 00	6.23e − 04∗
25	bidirectional	2	154	−6.39 ± 32.65	1.67 ± 22.25	2.53e + 00	1.19e − 02∗
30	bidirectional	2	142	−5.43 ± 33.10	2.27 ± 25.53	2.20e + 00	2.89e − 02∗
35	bidirectional	2	135	−5.93 ± 33.16	3.17 ± 24.70	2.55e + 00	1.12e − 02∗
40	bidirectional	2	135	−5.77 ± 31.69	0.02 ± 22.53	1.73e + 00	8.46e − 02
45	bidirectional	2	119	−7.92 ± 31.40	1.35 ± 20.62	2.69e + 00	7.64e − 03∗
50	bidirectional	2	102	−7.30 ± 30.98	−2.22 ± 28.78	1.21e + 00	2.26e − 01
55	bidirectional	2	92	−8.22 ± 31.10	−0.21 ± 26.09	1.89e + 00	6.01e − 02
60	bidirectional	2	86	−8.67 ± 30.48	2.66 ± 20.35	2.87e + 00	4.68e − 03∗
65	bidirectional	2	77	−6.87 ± 29.17	−1.20 ± 20.28	1.40e + 00	1.63e − 01
70	bidirectional	2	69	−5.87 ± 29.04	−1.62 ± 26.87	8.92e − 01	3.74e − 01
75	bidirectional	2	57	−5.17 ± 29.90	−3.20 ± 22.86	3.94e − 01	6.94e − 01
80	bidirectional	2	50	−2.19 ± 31.21	−6.68 ± 25.15	−7.92e − 01	4.30e − 01

**Figure 11:**
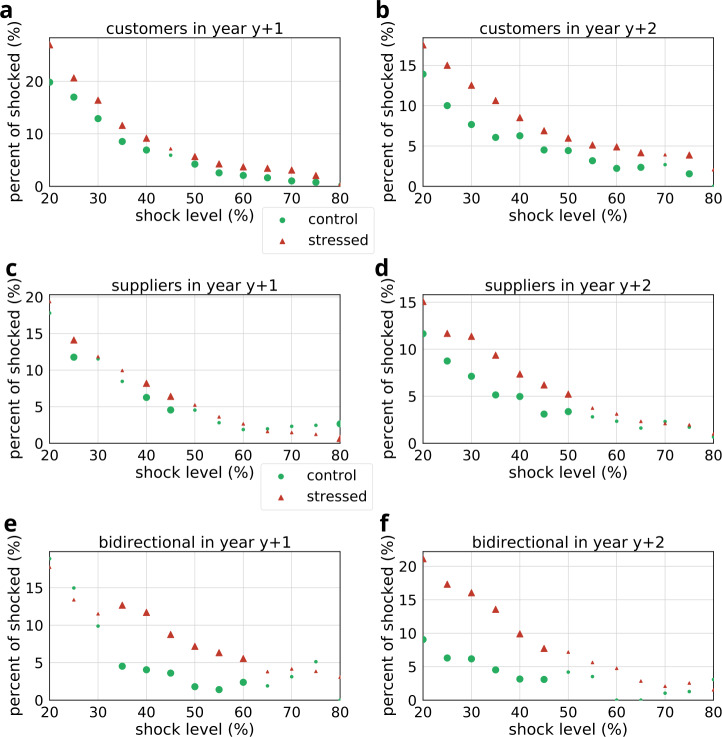
Comparison of the fraction of shocked companies within stressed and control groups at different shock levels. Large bullets indicate significant differences ($p<0.05$). Results are shown separately for stressed customers (**a**, **b**), suppliers (**c**, **d**) and bidirectional partners (**e**, **f**). (**a**, **c**, **e**) indicate fractions in the first year following the shock event ($y+1$), while (**b**, **d**, **f**) show fractions in the second year following the shock event ($y+2$). The corresponding statistics and sample sizes are listed in Table 7

**Figure 12:**
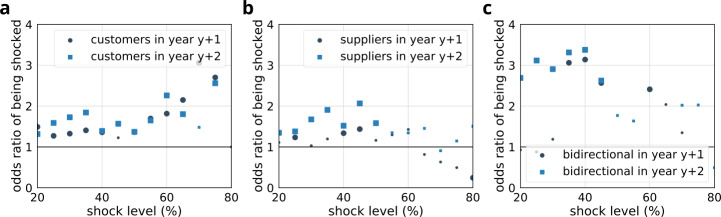
Odds ratio (stressed vs. control) of being shocked in case of customers (**a**), suppliers (**b**), and bidirectional partners (**c**) during the first and second years following the shock event. Large bullets indicate significant results ($p<0.05$). The corresponding statistics and sample sizes are listed in Table 7

Next we investigated whether stress results in future shocks and whether shocks show any cascading behavior (see Fig. 11 and Table 7). We calculated in years $y+1$ and $y+2$ the fraction of shocks within the stressed and control groups. Overall, consistent differences can be observed across all three stressed groups both in the first and second years following the shock. Most prominently customers with shocked suppliers are affected (see Fig. 11b, e.g., at 50% shock level $\chi ^{2}=5.48$, $p<0.019$). The corresponding odds-ratios are shown in Fig. 12.

**Table 7:** Frequency of shocked companies within stressed and control groups at different shock levels. Results are shown separately for stressed customers, suppliers and bidirectional partners in the first and second years following the original shock event. We provide sample sizes (*N*), frequencies, and the results of $\chi ^{2}$ tests with corresponding *p*-values

Stress level drop in sales (%)	Edge type	Year	*N*	Shocked (%) control	Shocked (%) stressed	$\chi ^{2}(\mathrm{Df} =1)$	*p*-value
20	customers	1	1488	26.95	19.82	4.75e + 01	5.48e − 12∗
25	customers	1	1419	20.65	16.98	1.35e + 01	2.37e − 04∗
30	customers	1	1396	16.40	12.89	1.53e + 01	9.11e − 05∗
35	customers	1	1335	11.61	8.54	1.61e + 01	5.94e − 05∗
40	customers	1	1244	9.16	6.91	9.79e + 00	1.75e − 03∗
45	customers	1	1131	7.16	5.92	3.11e + 00	7.78e − 02
50	customers	1	970	5.67	4.23	4.99e + 00	2.55e − 02∗
55	customers	1	819	4.27	2.56	9.58e + 00	1.97e − 03∗
60	customers	1	674	3.71	2.08	8.83e + 00	2.97e − 03∗
65	customers	1	552	3.44	1.63	1.13e + 01	7.77e − 04∗
70	customers	1	484	3.10	1.03	2.02e + 01	6.94e − 06∗
75	customers	1	386	2.07	0.78	8.40e + 00	3.76e − 03∗
80	customers	1	292	0.34	0.34	0.00e + 00	1.00e + 00
20	customers	2	1488	17.54	13.91	1.64e + 01	5.23e − 05∗
25	customers	2	1419	15.01	10.01	3.94e + 01	3.37e − 10∗
30	customers	2	1396	12.54	7.67	4.68e + 01	7.85e − 12∗
35	customers	2	1335	10.64	6.07	4.89e + 01	2.69e − 12∗
40	customers	2	1244	8.52	6.27	1.07e + 01	1.06e − 03∗
45	customers	2	1131	6.90	4.51	1.50e + 01	1.09e − 04∗
50	customers	2	970	5.98	4.43	5.48e + 00	1.93e − 02∗
55	customers	2	819	5.13	3.17	1.02e + 01	1.43e − 03∗
60	customers	2	674	4.90	2.23	2.21e + 01	2.60e − 06∗
65	customers	2	552	4.17	2.35	7.88e + 00	5.00e − 03∗
70	customers	2	484	3.93	2.69	2.85e + 00	9.16e − 02
75	customers	2	386	3.89	1.55	1.37e + 01	2.13e − 04∗
80	customers	2	292	2.06	0.00	–	–
20	suppliers	1	1399	19.37	17.80	2.36e + 00	1.24e − 01
25	suppliers	1	1360	14.12	11.77	7.25e + 00	7.08e − 03∗
30	suppliers	1	1292	11.84	11.53	1.21e − 01	7.28e − 01
35	suppliers	1	1206	9.95	8.46	3.47e + 00	6.25e − 02
40	suppliers	1	1086	8.20	6.26	6.92e + 00	8.53e − 03∗
45	suppliers	1	967	6.41	4.55	7.71e + 00	5.48e − 03∗
50	suppliers	1	861	5.23	4.53	9.67e − 01	3.25e − 01
55	suppliers	1	748	3.61	2.81	1.76e + 00	1.84e − 01
60	suppliers	1	641	2.65	1.87	2.12e + 00	1.45e − 01
65	suppliers	1	558	1.61	1.97	3.71e − 01	5.42e − 01
70	suppliers	1	476	1.47	2.31	1.49e + 00	2.22e − 01
75	suppliers	1	408	1.23	2.45	2.56e + 00	1.09e − 01
80	suppliers	1	302	0.66	2.65	4.62e + 00	3.16e − 02∗
20	suppliers	2	1399	15.08	11.65	1.60e + 01	6.34e − 05∗
25	suppliers	2	1360	11.69	8.75	1.47e + 01	1.24e − 04∗
30	suppliers	2	1292	11.38	7.12	3.54e + 01	2.68e − 09∗
35	suppliers	2	1206	9.37	5.14	4.42e + 01	2.93e − 11∗
40	suppliers	2	1086	7.37	4.97	1.32e + 01	2.84e − 04∗
45	suppliers	2	967	6.21	3.10	3.10e + 01	2.63e − 08∗
50	suppliers	2	861	5.23	3.37	9.14e + 00	2.51e − 03∗
55	suppliers	2	748	3.74	2.81	2.40e + 00	1.21e − 01
60	suppliers	2	641	3.12	2.34	1.71e + 00	1.91e − 01
65	suppliers	2	558	2.33	1.61	1.81e + 00	1.79e − 01
70	suppliers	2	476	2.10	2.31	9.31e − 02	7.60e − 01
75	suppliers	2	408	1.96	1.72	1.45e − 01	7.03e − 01
80	suppliers	2	302	0.99	0.66	5.03e − 01	4.78e − 01
20	bidirectional	1	265	17.74	18.87	2.22e − 01	6.38e − 01
25	bidirectional	1	254	13.39	14.96	4.95e − 01	4.82e − 01
30	bidirectional	1	243	11.52	9.88	7.40e − 01	3.90e − 01
35	bidirectional	1	221	12.67	4.53	3.39e + 01	5.70e − 09∗
40	bidirectional	1	222	11.71	4.05	3.35e + 01	7.24e − 09∗
45	bidirectional	1	194	8.76	3.61	1.48e + 01	1.18e − 04∗
50	bidirectional	1	167	7.19	1.80	2.75e + 01	1.58e − 07∗
55	bidirectional	1	142	6.34	1.41	2.49e + 01	6.20e − 07∗
60	bidirectional	1	126	5.56	2.38	5.46e + 00	1.94e − 02∗
65	bidirectional	1	105	3.81	1.91	2.04e + 00	1.53e − 01
70	bidirectional	1	96	4.17	3.12	3.44e − 01	5.57e − 01
75	bidirectional	1	78	3.85	5.13	2.64e − 01	6.08e − 01
80	bidirectional	1	65	3.08	0.00	–	–
20	bidirectional	2	265	21.13	9.06	4.69e + 01	7.41e − 12∗
25	bidirectional	2	254	17.32	6.30	5.23e + 01	4.78e − 13∗
30	bidirectional	2	243	16.05	6.17	4.09e + 01	1.58e − 10∗
35	bidirectional	2	221	13.57	4.53	4.19e + 01	9.63e − 11∗
40	bidirectional	2	222	9.91	3.15	3.32e + 01	8.36e − 09∗
45	bidirectional	2	194	7.73	3.09	1.39e + 01	1.90e − 04∗
50	bidirectional	2	167	7.19	4.19	3.73e + 00	5.35e − 02
55	bidirectional	2	142	5.63	3.52	1.87e + 00	1.72e − 01
60	bidirectional	2	126	4.76	0.00	–	–
65	bidirectional	2	105	2.86	0.00	–	–
70	bidirectional	2	96	2.08	1.04	1.01e + 00	3.15e − 01
75	bidirectional	2	78	2.56	1.28	1.01e + 00	3.14e − 01
80	bidirectional	2	65	1.54	3.08	5.16e − 01	4.73e − 01

### Effect of stress on the production network

**Figure 13:**
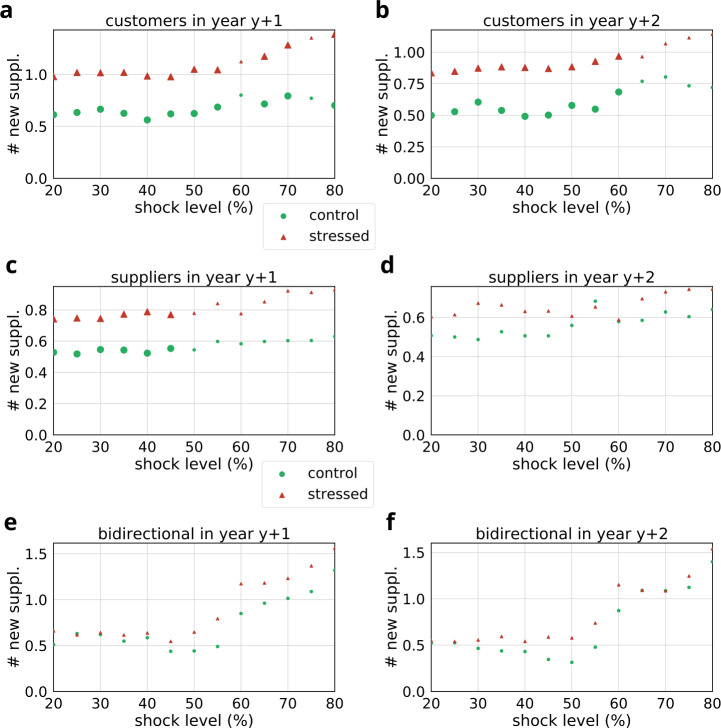
Comparison of the number of new suppliers of stressed and control groups at different shock levels. Large bullets indicate significant differences ($p<0.05$). Results are shown separately for stressed customers (**a**, **b**), suppliers (**c**, **d**) and bidirectional partners (**e**, **f**). (**a**, **c**, **e**) indicate the number of new suppliers in the first year following the shock event ($y+1$), while (**b**, **d**, **f**) show the number of new suppliers in the second year following the shock event ($y+2$). The corresponding statistics and sample sizes are listed in Table 8

**Figure 14:**
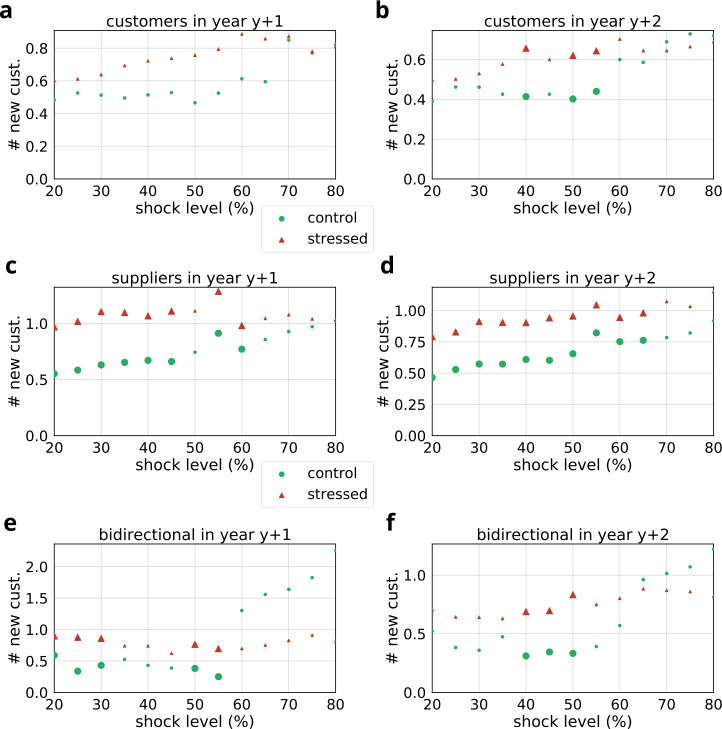
Comparison of the number of new customers of stressed and control groups at different shock levels. Large bullets indicate significant differences ($p<0.05$). Results are shown separately for stressed customers (**a**, **b**), suppliers (**c**, **d**) and bidirectional partners (**e**, **f**). (**a**, **c**, **e**) indicate the number of new customers in the first year following the shock event ($y+1$), while (**b**, **d**, **f**) show the number of new customers in the second year following the shock event ($y+2$). The corresponding statistics and sample sizes are listed in Table 9

Finally, we analyze the number of new links stressed companies initiate (see Figs. 13, 14 and Tables 8, 9). We show the number of new supplier links of stressed and control groups in Fig. 13. As expected, we find that both in the first and second years following the shock, stressed customers seek for more new suppliers (see Fig. 13a–b). For example, at 50% shock level customers seek in average for 1.05 new suppliers ($D=0.12$, $p<0.001$). Although some of the results are significant for stressed suppliers as well (see Fig. 13c), especially at lower stress levels (<50%) suppliers seem to initiate more new supplier relationships than their matched controls. These subtle differences diminish in the second year (all $p>0.05$, see Fig. 13d). Furthermore, we find no significant differences in the network activity of bidirectional partners.

**Table 8:** Comparison of the number of new suppliers of stressed and control groups at different shock levels. Results are shown separately for stressed customers, suppliers and bidirectional partners in the first and second years following the original shock event. We provide sample sizes (*N*), mean number of new customers, and the results of Kolmogorov–Smirnov tests with corresponding *p*-values

Stress level	Edge type	Year	*N*	Stressed	Control	D	*p*-value
20	customers	1	977	0.98	0.61	1.31e − 01	1.00e − 07∗
25	customers	1	925	1.02	0.63	1.41e − 01	2.21e − 08∗
30	customers	1	919	1.02	0.67	1.19e − 01	4.74e − 06∗
35	customers	1	866	1.02	0.63	1.50e − 01	6.29e − 09∗
40	customers	1	792	0.98	0.56	1.26e − 01	6.40e − 06∗
45	customers	1	718	0.98	0.62	1.16e − 01	1.34e − 04∗
50	customers	1	614	1.05	0.62	1.21e − 01	2.64e − 04∗
55	customers	1	509	1.04	0.69	1.20e − 01	1.32e − 03∗
60	customers	1	408	1.12	0.80	9.31e − 02	5.80e − 02
65	customers	1	338	1.18	0.72	1.15e − 01	2.21e − 02∗
70	customers	1	299	1.28	0.79	1.27e − 01	1.59e − 02∗
75	customers	1	236	1.35	0.77	1.06e − 01	1.42e − 01
80	customers	1	168	1.39	0.70	1.55e − 01	3.56e − 02∗
20	customers	2	977	0.84	0.50	1.36e − 01	2.62e − 08∗
25	customers	2	925	0.85	0.53	1.20e − 01	3.20e − 06∗
30	customers	2	919	0.87	0.60	1.15e − 01	9.60e − 06∗
35	customers	2	866	0.88	0.54	1.43e − 01	3.70e − 08∗
40	customers	2	792	0.88	0.49	1.64e − 01	9.95e − 10∗
45	customers	2	718	0.87	0.50	1.14e − 01	1.69e − 04∗
50	customers	2	614	0.88	0.58	8.63e − 02	2.06e − 02∗
55	customers	2	509	0.93	0.55	1.47e − 01	3.08e − 05∗
60	customers	2	408	0.97	0.68	1.18e − 01	7.01e − 03∗
65	customers	2	338	0.96	0.77	7.10e − 02	3.62e − 01
70	customers	2	299	1.07	0.80	8.03e − 02	2.91e − 01
75	customers	2	236	1.11	0.73	8.05e − 02	4.30e − 01
80	customers	2	168	1.14	0.72	1.07e − 01	2.90e − 01
20	suppliers	1	996	0.74	0.53	8.13e − 02	2.74e − 03∗
25	suppliers	1	928	0.75	0.52	9.48e − 02	4.71e − 04∗
30	suppliers	1	889	0.74	0.55	6.75e − 02	3.48e − 02∗
35	suppliers	1	825	0.77	0.54	8.12e − 02	8.65e − 03∗
40	suppliers	1	721	0.79	0.52	8.46e − 02	1.14e − 02∗
45	suppliers	1	635	0.77	0.55	8.03e − 02	3.32e − 02∗
50	suppliers	1	562	0.78	0.54	7.30e − 02	1.00e − 01
55	suppliers	1	483	0.84	0.60	5.38e − 02	4.86e − 01
60	suppliers	1	420	0.78	0.58	5.95e − 02	4.47e − 01
65	suppliers	1	378	0.85	0.60	5.82e − 02	5.45e − 01
70	suppliers	1	320	0.92	0.60	7.50e − 02	3.30e − 01
75	suppliers	1	273	0.91	0.60	5.86e − 02	7.37e − 01
80	suppliers	1	184	0.93	0.63	8.15e − 02	5.75e − 01
20	suppliers	2	996	0.60	0.51	2.51e − 02	9.12e − 01
25	suppliers	2	928	0.61	0.50	2.91e − 02	8.27e − 01
30	suppliers	2	889	0.67	0.49	4.72e − 02	2.74e − 01
35	suppliers	2	825	0.66	0.53	4.12e − 02	4.85e − 01
40	suppliers	2	721	0.63	0.51	3.74e − 02	6.93e − 01
45	suppliers	2	635	0.63	0.51	3.94e − 02	7.09e − 01
50	suppliers	2	562	0.61	0.56	1.42e − 02	1.00e + 00
55	suppliers	2	483	0.65	0.68	2.90e − 02	9.87e − 01
60	suppliers	2	420	0.59	0.58	9.52e − 03	1.00e + 00
65	suppliers	2	378	0.70	0.58	2.91e − 02	9.97e − 01
70	suppliers	2	320	0.73	0.63	2.19e − 02	1.00e + 00
75	suppliers	2	273	0.74	0.60	2.56e − 02	1.00e + 00
80	suppliers	2	184	0.74	0.64	4.35e − 02	9.95e − 01
20	bidirectional	1	159	0.66	0.51	8.81e − 02	5.70e − 01
25	bidirectional	1	154	0.62	0.63	8.44e − 02	6.44e − 01
30	bidirectional	1	142	0.64	0.62	7.04e − 02	8.75e − 01
35	bidirectional	1	135	0.61	0.55	5.19e − 02	9.94e − 01
40	bidirectional	1	135	0.64	0.58	4.44e − 02	9.99e − 01
45	bidirectional	1	119	0.55	0.44	8.40e − 02	7.97e − 01
50	bidirectional	1	102	0.65	0.44	1.47e − 01	2.21e − 01
55	bidirectional	1	92	0.79	0.49	1.52e − 01	2.38e − 01
60	bidirectional	1	86	1.17	0.85	8.14e − 02	9.40e − 01
65	bidirectional	1	77	1.18	0.96	7.79e − 02	9.75e − 01
70	bidirectional	1	69	1.23	1.01	1.01e − 01	8.73e − 01
75	bidirectional	1	57	1.37	1.09	8.77e − 02	9.82e − 01
80	bidirectional	1	50	1.56	1.32	6.00e − 02	1.00e + 00
20	bidirectional	2	159	0.55	0.52	1.89e − 02	1.00e + 00
25	bidirectional	2	154	0.54	0.53	1.30e − 02	1.00e + 00
30	bidirectional	2	142	0.56	0.47	4.23e − 02	1.00e + 00
35	bidirectional	2	135	0.59	0.44	4.44e − 02	9.99e − 01
40	bidirectional	2	135	0.54	0.43	3.70e − 02	1.00e + 00
45	bidirectional	2	119	0.59	0.34	8.40e − 02	7.97e − 01
50	bidirectional	2	102	0.58	0.31	1.57e − 01	1.63e − 01
55	bidirectional	2	92	0.74	0.48	7.61e − 02	9.55e − 01
60	bidirectional	2	86	1.15	0.87	4.65e − 02	1.00e + 00
65	bidirectional	2	77	1.09	1.09	1.30e − 02	1.00e + 00
70	bidirectional	2	69	1.09	1.09	2.90e − 02	1.00e + 00
75	bidirectional	2	57	1.25	1.12	1.05e − 01	9.14e − 01
80	bidirectional	2	50	1.54	1.40	1.20e − 01	8.69e − 01

**Table 9:** Comparison of the number of new customers of stressed and control groups at different shock levels. Results are shown separately for stressed customers, suppliers and bidirectional partners in the first and second years following the original shock event. We provide sample sizes (*N*), mean number of new suppliers, and the results of Kolmogorov–Smirnov tests with corresponding *p*-values

Stress level	Edge type	Year	*N*	Stressed	Control	D	*p*-value
20	customers	1	977	0.60	0.48	3.38e − 02	6.33e − 01
25	customers	1	925	0.61	0.53	2.49e − 02	9.38e − 01
30	customers	1	919	0.64	0.51	3.48e − 02	6.33e − 01
35	customers	1	866	0.69	0.49	4.04e − 02	4.79e − 01
40	customers	1	792	0.72	0.51	4.42e − 02	4.22e − 01
45	customers	1	718	0.74	0.53	3.90e − 02	6.46e − 01
50	customers	1	614	0.76	0.47	5.54e − 02	3.03e − 01
55	customers	1	509	0.79	0.53	4.91e − 02	5.72e − 01
60	customers	1	408	0.89	0.61	5.39e − 02	5.94e − 01
65	customers	1	338	0.86	0.59	6.21e − 02	5.32e − 01
70	customers	1	299	0.87	0.85	5.02e − 02	8.47e − 01
75	customers	1	236	0.78	0.77	2.54e − 02	1.00e + 00
80	customers	1	168	0.81	0.82	2.38e − 02	1.00e + 00
20	customers	2	977	0.50	0.39	4.40e − 02	3.00e − 01
25	customers	2	925	0.50	0.46	3.46e − 02	6.38e − 01
30	customers	2	919	0.53	0.46	3.70e − 02	5.56e − 01
35	customers	2	866	0.58	0.43	5.20e − 02	1.93e − 01
40	customers	2	792	0.66	0.41	7.45e − 02	2.46e − 02∗
45	customers	2	718	0.60	0.43	5.85e − 02	1.71e − 01
50	customers	2	614	0.62	0.40	8.63e − 02	2.06e − 02∗
55	customers	2	509	0.64	0.44	9.43e − 02	2.16e − 02∗
60	customers	2	408	0.70	0.60	3.19e − 02	9.86e − 01
65	customers	2	338	0.65	0.59	1.18e − 02	1.00e + 00
70	customers	2	299	0.65	0.69	2.01e − 02	1.00e + 00
75	customers	2	236	0.67	0.73	4.24e − 02	9.84e − 01
80	customers	2	168	0.69	0.72	4.17e − 02	9.99e − 01
20	suppliers	1	996	0.97	0.55	1.14e − 01	4.21e − 06∗
25	suppliers	1	928	1.02	0.58	1.21e − 01	2.63e − 06∗
30	suppliers	1	889	1.10	0.63	1.16e − 01	1.29e − 05∗
35	suppliers	1	825	1.10	0.65	1.04e − 01	2.53e − 04∗
40	suppliers	1	721	1.07	0.67	9.57e − 02	2.70e − 03∗
45	suppliers	1	635	1.11	0.66	9.61e − 02	5.68e − 03∗
50	suppliers	1	562	1.11	0.74	7.65e − 02	7.45e − 02
55	suppliers	1	483	1.29	0.91	8.90e − 02	4.35e − 02∗
60	suppliers	1	420	0.98	0.77	1.00e − 01	2.99e − 02∗
65	suppliers	1	378	1.04	0.86	9.52e − 02	6.48e − 02
70	suppliers	1	320	1.08	0.93	9.06e − 02	1.44e − 01
75	suppliers	1	273	1.04	0.97	6.96e − 02	5.24e − 01
80	suppliers	1	184	1.03	1.02	7.07e − 02	7.49e − 01
20	suppliers	2	996	0.79	0.47	9.74e − 02	1.56e − 04∗
25	suppliers	2	928	0.83	0.53	9.48e − 02	4.71e − 04∗
30	suppliers	2	889	0.91	0.57	9.79e − 02	3.98e − 04∗
35	suppliers	2	825	0.91	0.57	8.73e − 02	3.72e − 03∗
40	suppliers	2	721	0.90	0.61	8.88e − 02	6.80e − 03∗
45	suppliers	2	635	0.94	0.60	9.61e − 02	5.68e − 03∗
50	suppliers	2	562	0.96	0.66	1.25e − 01	3.22e − 04∗
55	suppliers	2	483	1.05	0.82	8.90e − 02	4.35e − 02∗
60	suppliers	2	420	0.94	0.75	1.10e − 01	1.29e − 02∗
65	suppliers	2	378	0.98	0.76	1.14e − 01	1.50e − 02∗
70	suppliers	2	320	1.07	0.78	1.00e − 01	8.15e − 02
75	suppliers	2	273	1.03	0.82	8.79e − 02	2.42e − 01
80	suppliers	2	184	1.15	0.92	9.24e − 02	4.13e − 01
20	bidirectional	1	159	0.90	0.59	1.64e − 01	2.83e − 02∗
25	bidirectional	1	154	0.88	0.34	2.27e − 01	6.72e − 04∗
30	bidirectional	1	142	0.86	0.43	1.62e − 01	4.81e − 02∗
35	bidirectional	1	135	0.74	0.53	1.19e − 01	3.00e − 01
40	bidirectional	1	135	0.74	0.43	1.56e − 01	7.62e − 02
45	bidirectional	1	119	0.62	0.39	1.26e − 01	3.02e − 01
50	bidirectional	1	102	0.77	0.38	2.06e − 01	2.63e − 02∗
55	bidirectional	1	92	0.70	0.25	2.17e − 01	2.56e − 02∗
60	bidirectional	1	86	0.70	1.30	1.05e − 01	7.37e − 01
65	bidirectional	1	77	0.75	1.56	1.82e − 01	1.57e − 01
70	bidirectional	1	69	0.83	1.64	1.74e − 01	2.49e − 01
75	bidirectional	1	57	0.91	1.82	2.28e − 01	1.03e − 01
80	bidirectional	1	50	0.82	2.26	1.20e − 01	8.69e − 01
20	bidirectional	2	159	0.70	0.52	1.45e − 01	7.17e − 02
25	bidirectional	2	154	0.64	0.38	1.30e − 01	1.49e − 01
30	bidirectional	2	142	0.64	0.36	1.55e − 01	6.61e − 02
35	bidirectional	2	135	0.63	0.47	1.19e − 01	3.00e − 01
40	bidirectional	2	135	0.69	0.31	1.85e − 01	1.93e − 02∗
45	bidirectional	2	119	0.70	0.34	1.76e − 01	4.90e − 02∗
50	bidirectional	2	102	0.83	0.33	2.16e − 01	1.72e − 02∗
55	bidirectional	2	92	0.75	0.39	1.41e − 01	3.19e − 01
60	bidirectional	2	86	0.80	0.57	1.28e − 01	4.85e − 01
65	bidirectional	2	77	0.88	0.96	1.04e − 01	8.04e − 01
70	bidirectional	2	69	0.87	1.01	5.80e − 02	1.00e + 00
75	bidirectional	2	57	0.86	1.07	1.58e − 01	4.80e − 01
80	bidirectional	2	50	0.82	1.22	6.00e − 02	1.00e + 00

Next we present results in a similar way for new customer links of stressed and control groups in Fig. 14. In the first year follow-up there is no significant difference between stressed and control customers (see Fig. 14a). However, in the second year follow-up stressed customers (between 40% and 60% stress level) are initiating more relationships with new customers than matched controls (see Fig. 14b). We find the largest significant differences consistently nearly at all shock levels between the activity of stressed and control suppliers (see Fig. 14c–d) in both the first and second years following the initial shock. In case of bidirectional partners, the results show noticeable differences between groups at certain shock levels (<60%). Taken together, the results indicate a more intense activity in the stress group than in the control group in terms of new links.

Finally, our results indicate that 14.8% of the new supplier links initiated in year $y+1$ of stressed customers have a matching industry label to the shocked neighbor. The same percentage is 19.8% in year $y+2$. The similar quantities for stressed suppliers looking for new customers are 18.3% ($y+1$) and 24.1% ($y+2$), respectively. These overlaps are considerably large and suggest that new links are often “replacements” and stressed partners quickly react to shocks within the production network.

## Discussion and conclusion

The current study explores the impact of shocks in the production network. We examined a unique dataset containing temporal information on companies for an 8 year period from Slovenia. We found that economic partners of shocked companies have a higher chance of going bankrupt, even if they do not show signs of performance loss at the time of the shocking event. These results suggest that not only sales shocks, but the shock induced stress is related to bankruptcy. It is important to highlight that besides these, other factors including the sales performance, the number of customers, and the industry sector significantly impact the likelihood of future bankruptcy as seen in our logistic regression based results. These findings are in line with previous reports [2, 22].

We hypothesized that stressed companies experience loss in performance in the follow-up years. Our results support this hypothesis. Specifically, we found that stressed customers show a significant drop in sales compared to control companies. However, compared to the stressed customers, suppliers appear to be more resilient to the shock induced stress in terms of sales performance. Bidirectional relationships are more stable in the first year follow-up, while their performance seems to be heavily impacted in the second year. This might reflect a temporal shift in the impact of shock on mutually dependent partnerships. We note that bidirectional partnerships represent a complex form of economical interactions and give place to the interplay of multiple network effects. Detailed analysis of the bidirectional partnerships is out of the scope of the current study, however the increased likelihood of being shocked in this category with the above mentioned performance stability raises important questions for future experiments.

The result that stress frequently leads to shock, makes it likely that there is a shock spill over to other neighbors in the network. These results are in line with [2], where the authors examined the customers of suppliers hit by a natural disaster, and reported that firm-level shocks propagate within production networks.

Regarding the stress induced reconfiguration of the network we hypothesized that the tense urges the stressed party to replace the problematic partner. More specifically, we expected that companies’ new partnership seeking would vary according to their relationship with the shocked neighbor. In line with our expectations customers of the shocked node initiated more new partnerships with suppliers, and suppliers improved the numbers of their customers consistently for at least two years. Furthermore, we found that these new links often share the industry sector of the initially shocked node and hence may be potential replacements. Surprisingly, in case of the suppliers we have observed an emphasised seek for new suppliers at lower stress levels. This might be associated with their above presented stress resilience.

### Limitations and future work

While the data and our analyses presents various aspects of the production network, we must note their limitations. Foremost, since no performance indicators were available for foreign companies, we investigated the customer-supplier relations within a single country, which decreases the generalizability of the presented results. Furthermore, since companies are required to send all funds over 50k EUR via the TARGET2 system, the data covers all of the large transactions, but not necessarily all of the smaller transactions that are less than 50k EUR. Some part of the customer-supplier relations may remain uncovered which can lead to an overall underestimation of the effects of stress. Throughout the paper we consider all industry sectors other than governmental and financial services to be part of the production network. Some of the companies within our analyses may not strictly belong to the production network. More information on the companies activities could provide a more precise view of the network itself. Surprisingly, a few of the reported results potentially show trends with the shock threshold, e.g., there is an increasing trend in Fig. 10. Future analyses would be required to validate if these are actual signals or artifacts within the data analyzed.

The data studied is right after the 2008 global financial crisis and therefore we consider this the broad, implicit cause of the disruptions within the nation’s economy. Throughout the analyses we study sales drops that are strong indicators of “shocks”. However, we do not have explicit information on the specific root cause of shocks.

In this paper we employ a time sensitive, matched pairs quasi-experimental design and the presented analyses correct for confounders such as the company’s industry sector, current sales, number of customer and supplier links. There can be additional factors to consider and follow-up analyses with different matching criteria can lead to new discoveries. For example, one could restrict the control group to have at least one connection to the industry category of the shocked company. Although we control for several potential confounders, richer datasets may allow to correct for additional variables other than the ones considered in this study.

Although our results show that sudden sales losses, shocks, heavily impact their immediate environment, some companies seem to be resilient against stress. Future investigation would be needed to identify potential protective factors that contribute to stress resilience.

## Data Availability

All presented data were used under license for the current study and not available publicly for secondary analysis. Data are potentially available from the authors upon reasonable request and with permission of the National Bank of Slovenia.

## Footnotes

1 https://en.wikipedia.org/wiki/Financial_crisis_of_2007-2008

2 https://en.wikipedia.org/wiki/TARGET2

3 https://www.stat.si/statweb/en/methods/classifications
